# Pharmacokinetic modelling of orally administered cannabidiol and implications for medication control in horses

**DOI:** 10.3389/fvets.2023.1234551

**Published:** 2023-08-09

**Authors:** Fabienne Eichler, Błażej Poźniak, Marc Machnik, Ina Schenk, Anke Wingender, Natalie Baudisch, Mario Thevis, Wolfgang Bäumer, Christoph Lischer, Anna Ehrle

**Affiliations:** ^1^Equine Clinic, Veterinary Hospital Freie Universität Berlin, School of Veterinary Medicine, Freie Universität Berlin, Berlin, Germany; ^2^Department of Pharmacology and Toxicology, Faculty of Veterinary Medicine, Wrocław University of Environmental and Life Sciences, Wrocław, Poland; ^3^Center for Preventive Doping Research, Institute of Biochemistry, German Sport University Cologne, Cologne, Germany; ^4^Institute of Pharmacology and Toxicology, School of Veterinary Medicine, Freie Universität Berlin, Berlin, Germany

**Keywords:** CBD, cannabinoids, doping, drug control, equine, Monolix, PK, NLME model

## Abstract

Cannabidiol (CBD) products gain increasing popularity amongst animal owners and veterinarians as an alternative remedy for treatment of stress, inflammation or pain in horses. Whilst the use of cannabinoids is banned in equine sports, there is limited information available concerning CBD detection times in blood or urine. The aim of this study was to determine the pharmacokinetic properties of CBD following oral administration in the horse to assist doping control laboratories with interpreting CBD analytical results. Part 1: dose escalation study: Single oral administration of three escalating doses of CBD paste (0.2 mg/kg, *n* = 3 horses; 1 mg/kg, *n* = 3; 3 mg/kg, *n* = 5) with >7 days wash-out periods in between. Part 2: multiple dose study: oral administration of CBD paste (3 mg/kg, *n* = 6) twice daily for 15 days. Multiple blood and urine samples were collected daily throughout both studies. Following study part 2, blood and urine samples were collected for 2 weeks to observe the elimination phase. Concentrations of CBD, its metabolites and further cannabinoids were evaluated using gas-chromatography/tandem-mass-spectrometry. Pharmacokinetic parameters were assessed via two approaches: population pharmacokinetic analysis using a nonlinear mixed-effects model and non-compartmental analysis. AUC_0–12 h_ and *C*_max_ were tested for dose proportionality. During the elimination phase, the CBD steady-state urine to serum concentration ratio (Rss) was calculated. Oral CBD medication was well-tolerated in horses. Based on population pharmacokinetics, a three-compartment model with zero-order absorption most accurately described the pharmacokinetic properties of CBD. High volumes of distribution into peripheral compartments and high concentrations of 7-carboxy-CBD were observed in serum. Non-compartmental analysis identified a *C*_max_ of 12.17 ± 2.08 ng/mL after single administration of CBD (dose: 3 mg/kg). AUC_0–12 h_ showed dose proportionality, increase for *C*_max_ leveled off at higher doses. Following multiple doses, the CBD terminal half-life was 161.29 ± 43.65 h in serum. Rss was 4.45 ± 1.04. CBD is extensively metabolized and shows high volumes of tissue distribution with a resulting extended elimination phase. Further investigation of the potential calming and anti-inflammatory effects of CBD are required to determine cut-off values for medication control using the calculated Rss.

## 1. Introduction

Medical cannabis and its extracted cannabinoids are used for the treatment of chronic pain, spasticity, epilepsy and anxiety in humans, and have been gaining popularity for similar indications in veterinary medicine in recent years (1–5). The cannabinoids most commonly known are cannabidiol (CBD), cannabidiolic acid (CBDA) and ∆9-tetrahydrocannabinol (THC) (6). CBD interacts with the CB_1_- and CB_2_ receptors of the endogenous endocannabinoid system and is described to have anti-inflammatory, relaxing, anti-convulsant and anxiolytic effects, whilst THC is the main agent responsible for the psychotropic characteristics of cannabis (7–14).

Pharmacokinetic studies in healthy dogs and cats, as well as clinical studies investigating the treatment of osteoarthritis, canine epilepsy and canine atopic dermatitis have confirmed positive outcomes with little side effects following the oral administration of CBD oil or paste (5, 15–23). Initial scientific reports of CBD application in horses described the treatment of mechanical allodynia, second intention wound healing and treatment for stereotypic behavior such as crib-biting (24–27). Subsequent studies started to analyze the pharmacokinetic properties of cannabinoids in horses and some studies reported positive therapeutic effects particularly for the treatment of chronic degenerative pain in horses (28–35).

Due to their potential analgesic and psychotropic properties, natural and synthetic cannabinoids are on the list of banned substances in most national and international equine sports associations including the FEI (Fédération Equestre Internationale) (36, 37). CBD and CBDA were moved to the FEI’s list of controlled medications as specified substances in 2022 (36). The lipophilic properties of CBD and other cannabinoids can lead to the accumulation in organs and adipose tissue (5, 10, 38). The detection of synthetic cannabinoids in the context of doping control in horses has been described. There are, however, no further reports for detection times of CBD (36, 37, 39).

The aim of this study was to investigate the pharmacokinetic properties of CBD in horses following oral administration of a CBD containing paste, and to use the results for the interpretation of analytical findings following medication control in equestrian sports. The authors hypothesized that cannabinoids would have long retention times in equine biological matrices.

## 2. Materials and methods

### 2.1. Animals

Six Haflinger × Warmblood cross horses, including three mares and three stallions were included in the study. Mares and stallions were stabled in separate barns where the mares were kept in paddock boxes. All horses had *ad libitum* access to water, were fed hay and mineral feed and were led to pasture for 8 hours a day. The study was reviewed and approved by the competent authority for licensing and notification procedures for animal experiments (LAVG) in Brandenburg, Germany (AZ: 2347-12-2021).

### 2.2. CBD product

A paste containing 55% CBD (2,750 mg) and <0.2% THC (TAMACAN XL 55%^®^, 5,000 mg, Herosan healthcare GmbH, Austria) was used for oral medication. Further ingredients included naturally occurring phytocannabinoids, medium-chain triglyceride coconut oil, terpenes, flavonoids and beeswax. CBD and THC contents were analyzed and confirmed by an independent and internationally accredited anti-doping laboratory (Institute of Biochemistry, German Sport University Cologne, Cologne, Germany).

### 2.3. Dose escalation study

Initially, the CBD paste was administered in single escalating doses during three individual trials (trial 1: 0.2 mg/kg BWT, *n* = 3 horses; trial 2: 1 mg/kg, *n* = 3; trial 3: 3 mg/kg, *n* = 6). For better acceptance, the paste was inserted into a treat. There was a minimum washout period of 7 days in between trials. Prior to each trial, a physical examination was performed and a jugular vein catheter was aseptically placed. Blood samples were collected at the time points 0, 0.5, 1, 2, 4 and 12 hours (h) post medication for analysis of cannabinoid concentrations and for complete blood count (CBC; Diatron Abacus Junior 30 hematology analyser). Spontaneous urine samples were additionally collected at 2 and 12 h to be analyzed for cannabinoids. A repeated physical examination was performed between the time points 2–4 h following medication and horses were closely monitored for any signs of adverse reaction.

### 2.4. Multiple dose study

After a 25-day washout period, horses (*n* = 6) were administered oral CBD paste (3 mg/kg) every 12 hours for 15 days. Physical examinations were performed daily. Blood samples were obtained every day following oral medication at 2 and 11.5 h. CBC was performed daily at 2 h post administration (p.a.), and both the 2 and 11.5 h samples were analyzed for cannabinoid content. One spontaneous urine sample for cannabinoid analysis was collected from each horse between the time points 8–11.5 h. Serum kidney and liver biomarkers [blood urea nitrogen (BUN), creatinine (CREA), gamma-glutamyltransferase (GGT), glutamic oxaloacetic transaminase (GOT)] were assessed once a week (Fujifilm DRI-CHEM NX500i dry-chemistry analyser).

Following the final CBD oral application in the morning of day 15, blood samples were obtained at the time points 0, 0.5, 1, 2, 4 and 12 h and urine samples close to scheduled time points at 2 and 12 h for accurate monitoring of the drug elimination phase. Over the following 4 days (days 16–19), blood and urine samples were taken every 24 h and subsequently every 36–48 h until day 33. CBC and serum kidney and liver biomarkers were assessed 1 week after trial end.

### 2.5. Cannabinoid analysis

Serum and urine samples were frozen and stored at −20°C until further processing. Quantitative analysis for cannabinoid concentrations was performed at an independent and internationally accredited anti-doping laboratory (Institute of Biochemistry, German Sport University Cologne, Cologne, Germany). All samples were analyzed by gas chromatography/tandem mass spectrometry (GC/MS/MS) for the presence of CBD, CBDA, cannabidivarin (CBDV), cannabigerol (CBG), THC, 11-nor-9-carboxy-∆9-tetrahydrocannabinol (COOH-THC) and 11-hydroxy-∆9-tetrahydrocannabinol (OH-THC). 7-carboxy-cannabidiol (COOH-CBD) and 7-hydroxy-cannabidiol (OH-CBD) were additionally assessed in serum and urine, respectively. Additional information on the sample preparation/extraction and instrumental conditions that were used in this study are summarized in the Supplementary material.

For the validation of analytical methods, parameters including precision, accuracy, selectivity, robustness, linearity, the lower limit of detection (LLOD), lower limit of quantification (LLOQ) and stability were determined. For selectivity, product ion scans were compared with spectra from the literature (40) or from spectra libraries. Three diagnostic product ions of each analyte were included in the acquisition method. Ten blank samples of each specimen (serum and urine) were prepared as described above and tested for interfering peaks at the expected retention time of the analytes. The samples showed no significant signals that could be attributed to the analytes. It was therefore concluded that the selectivity criteria of the employed method were met.

To evaluate the robustness of the method, 10 different samples of each specimen were spiked with 5 ng/mL of each cannabinoid, prepared and analyzed on two consecutive days. Potential effects of the different sample matrices (e.g., biological background interferences, specific gravity and pH differences, different horse characteristics like gender, race and age, potential haemolysis and analytical system performance) on the detectability (reproducibility of ion ratios, peak shape, signal intensity, signal-to-noise ratio and retention times) of each cannabinoid were controlled and documented. All samples showed signals for each analyte with reproducible signal intensities and ion ratios. Relative retention time shifts were within acceptable ranges <0.8% for all tested cannabinoids.

Linearity for all tested cannabinoids was examined by a series of spiked samples at 10 different concentrations in serum and urine over a concentration range considering the expected concentrations in p.a. samples. Area ratios of analyte and internal standard (*y*) were plotted against the analyte concentration (*x*) and a calibration curve (*y* = *ax* + *b*) was generated by linear least square regression with a weighting factor of 1/*x* or 1/*x*^2^ (Thermo Scientific Excalibur software version 4.0). The spiked concentration (theoretical concentration) was compared to the calculated concentration (measured concentration) of each calibrator. Correlation factors (*R*^2^) were >0.98 for all calibration curves and measured concentrations were within the acceptance range of 85%–115% of the theoretical concentration for all cannabinoids.

A signal-to-noise ratio of ≥3 for the most abundant ion transition (quantifier ion) was used to determine the LLOD and a signal-to-noise ratio of ≥9 for the LLOQ in urine and serum. The LLOQ was verified by a six-fold determination of the estimated level to obtain the respective precision. The requirement for acceptance of the LLOQ was a coefficient of variation (CV) below 20%. Precisions were determined using 18 quality control (QC) samples which were spiked at low, medium and high concentrations quantified within 1 day (*n* = 6) and on three separate occasions (*n* = 6 + 6 + 6). The CV was established by 6 (intra-day precision) and 18 samples (inter-day precision). Respective concentrations of the QC samples and precisions for the four relevant cannabinoids in this study (CBD, CBDA, 7-COOH-CBD and 7-OH-CBD) are listed in Table 1. For the validation of the accuracy, QC samples (*n* = 6) each spiked at low, medium and high concentrations were quantified with a calibration curve. The means of measured values were compared with the theoretical values. Accuracies are expressed as relative errors (RE).

**Table 1:** Validation results of the relevant cannabinoids in the present study.

Canna binoid	Matrix	LLOD (ng/mL)	LLOQ (ng/mL)	Intra-day precision CV (%) at 0.5/5.0/50 ng/mL	Inter-day precision CV (%) at 0.5/5.0/50 ng/mL	Accuracy RE (%) at 0.5/5.0/50 ng/mL	Stability [%]
CBD	Serum Urine	0.1 0.1	0.2 0.2	9.4/3.9/1.6 4.4/5.1/5.4	6.9/3.7/4.1 5.7/4.4/5.0	9.9/1.6/6.5 −0.4/−2.5/−6.6	63 83
CBDA	Serum Urine	0.1 0.1	0.5 0.5	22.4/13.6/26.1 19.9/9.3/9.9	25.5/16.7/19.7 20.3/15.5/16.0	−2.8/−15.0/−12.4 −19.4/−12.6/−7.1	51 45
7-COOH-CBD	Serum	0.1	0.2	12.5/5.8/6.7	12.5/6.1/4.2	1.4/2.7/−2.5	45
7-OH-CBD	Urine	0.1	0.2	10.0/4.9/3.9	9.4/11.4/6.6	2.5/−6.2/−3.7	79

LLOD, lower limit of detection; LLOQ, lower limit of quantification; CV, coefficient of variation; RE, relative error; CBD, cannabidiol; CBDA, cannabidiolic acid; 7-COOH-CBD, 7-carboxy-cannabidiol; 7-OH-CBD, 7-hydroxy-cannabidiol.

The stability was assessed by means of 12 serum and urine samples, each fortified with the tested cannabinoids at 5 ng/mL. One set of samples (6 serum and 6 urine) were prepared and analyzed on day 1, whereas the other spiked sample sets (6 serum and 6 urine) were stored at −20°C for 100 days and then quantified using freshly prepared calibrators. Stability was expressed as percentage ratio of the mean concentration at day 100 and the mean concentration at day 1.

Table 1 summarizes the resulting LLODs, LLOQs, precisions, accuracies and stabilities that were validated for each matrix and each compound.

### 2.6. Pharmacokinetic analysis

#### 2.6.1. Non-compartmental analysis

Non-compartmental analysis (NCA) was performed on serum CBD and its metabolites using PKanalix^™^ 2021R2 (MonolixSuite^™^ 2021R2, Lixoft, Antony, France). For the dose escalation study, the area under the curve from the first to the last sampling time point (AUC_0–12 h_), and value and time of maximum serum concentration (*C*_max_ and *t*_max_) were calculated for CBD, 7-OH-CBD and 7-COOH-CBD and summarized as means and standard deviations (SD). The ratio of the AUC_0–12 h_ for 7-OH-CBD/CBD and 7-COOH-CBD/CBD was additionally calculated. For the multiple dose study, the terminal half-life was determined for CBD and 7-COOH-CBD based on the last six time points.

#### 2.6.2. Population pharmacokinetic analysis via a nonlinear mixed-effects model

To evaluate further pharmacokinetic parameters, serum CBD data was used to build a nonlinear mixed-effects model (NLME) applying the stochastic approximation expectation maximization (SAEM) algorithm with Monolix^™^ 2021R2. All CBD values from the dose escalation and the multiple dose studies were combined and fed into the software. The mean of the full posterior distribution was used to determine individual pharmacokinetic parameters. A mathematical model was written based on previous descriptions (41) with further refinements for veterinary purposes (42, 43):


$$y_{ij}=F\left(\varphi_{i},t_{ij}\right)+G\left(\varphi_{i},t_{ij},\beta \right)\times \varepsilon_{ij}$$

$$\varepsilon_{ij}\sim N\left(0,\sigma^{2}\right),\varphi_{i}=h\left(\mu ,\eta_{i},\beta_{i}\right)$$

$$\varphi_{i}=\mu \times e^{\eta_{i}},\eta_{i}\sim N\left(0,\Omega ,\omega^{2}\right)$$

$$i=1,\ldots ,N,j=1,\ldots ,n_{i}$$

*i* stands for each single individual with *N* being the sum of all individuals. Sample times from 1 to *n_i_* are described by *j*. *y_ij_* is the CBD concentration observed per individual at time *t_ij_*. The function *F*(*φ_i_*,*t_ij_*) predicts the individual concentration through parameter vector *φ_i_* at timepoint *t_ij_*. The associated residual error model G (*φ_i_*,*t_ij_*,*β*) contains the covariate *β* and is multiplied by the independent random variable $\varepsilon_{ij},$ which has a standard normal distribution including mean 0 and variance *σ*^2^. The parameter vector *φ_i_* was modelled as a function (*h*) of the mean population parameter *μ* with random variable *η_i_* describing the individual variability and individual covariate *β_i_*. A normal distribution of *η_i_* with mean value 0, variance-covariance matrix Ω and variance *ω*^2^ is assumed, leading to a log-normal distribution of individual parameters *φ_i_*.

The final model was described by three compartments and zero-order absorption. The data set included oral administration only; therefore, the assessment of clearance (Cl) and volumes of distribution (V) was biased by the unknown bioavailability (F). Model parameters include the duration of the zero-order absorption (Tk0), systemic clearance (Cl/F), volume of distribution of a central (V1/F) and two peripheral (V2/F, V3/F) compartments, and intercompartmental clearances (Q2, Q3). Predicted *C*_max_ and *t*_max_ values were obtained from the tables generated for the individual predicted curves.

*C*_max_ were used to calculate the accumulation ratio (AR):


$$\mathrm{AR}=\frac{C_{\max\_\mathrm{multipledose}}}{C_{\max\_\mathrm{singledose}}}$$

##### 2.6.2.1. Parameter correlation estimates

To identify correlations between parameters which could aid model performance, scatterplots of *η_i_* versus *η_i_*-values for pharmacokinetic parameter estimates’ pairs and the Pearson’s correlation coefficient were evaluated. A *t*-test was performed to test statistical significance, defined as a *p*-value of <0.05. The obtained samples from the posterior distribution at the last SAEM iteration and the empirical Bayes estimates (EBEs) were assessed for parameter correlation, with the EBEs considered less relevant (43, 44). Correlations which fitted the defined selection criteria (see section 2.6.2.2 Model evaluation) were added to the final model.

##### 2.6.2.2. Model evaluation

Numerical and graphical outputs (standard goodness-of-fit criteria, GOF) were used to evaluate the quality of the model (43, 44). To assess the SAEM algorithm, the stability of the parameter search and precision of the parameter estimates were examined for convergence through the relative standard error of the estimate (determined in the Fisher information matrix). Overparameterization was checked through the condition number of the eigenvalues. For graphical information, assessments were performed on individual observations vs. predictions, individual weighted residuals (IWRES), normalized predicted distribution errors (NPDE), visual predictive check (VPC) and individual fits. Distribution of the individual parameters and standardized random effects were examined through histograms and quantile-quantile plots. The random effects were evaluated for normal distribution using the Shapiro–Wilk test and the full posterior distribution of random effects and residuals. Models which performed satisfactorily were further inspected for precision of their respective parameter estimates and corrected Bayesian information criterion (BICc), before settling on a final model.

##### 2.6.2.3. Addition of covariates

The horses’ bodyweight was considered as a continuous covariate. The impact on model performance was assessed through the Pearson’s correlation coefficient, Wald test and analysis of variance (threshold: *p*-value <0.05).

#### 2.6.3. Dose proportionality

Pharmacokinetic parameters AUC_0–12 h_ and *C*_max_ for CBD were tested for dose proportionality using the individual values obtained from NCA and population pharmacokinetic analysis during the dose escalation study. Individual values were pooled for each parameter and fitted into a previously described power model (45, 46). Pharmacokinetic parameters (*y*) were log-transformed to apply a linear regression approach with dose as a covariate:


log(y)=μ+β×log(dose)

The closer the *β* value is to 1, the more proportionally doses are aligned.

Additionally, the individual pharmacokinetic parameters were log-transformed and dose-normalized to test for significant differences (defined as *p*-value <0.05) between each trial using an analysis of variance (ANOVA) with a post-hoc Tukey test (Statistica 13, TIBCO, Palo Alto, CA, United States).

### 2.7. Application to medication control

Medication control in equestrian sports is either performed in urine or blood samples. To draw conclusions about the levels in urine from an existing blood sample of a medication, Toutain and Lassourd recommend estimating the steady-state urine to serum concentration ratio (Rss) of a potential drug (47). The concentrations of CBD in urine (Css_urine_) and serum (Css_serum_) were used to calculate the Rss during the elimination phase of the multiple dose study (pseudo-equilibrium condition) (47, 48):


$$\mathrm{Rss}=\frac{{\mathrm{Css}}_{\mathrm{urine}}}{{\mathrm{Css}}_{\mathrm{serum}}}$$

## 3. Results

### 3.1. Horses

The horses’ ages ranged from 3 to 16 years (median = 11 years) and the body weight was 488 ± 55 kg. One horse developed a jugular vein thrombophlebitis during the third trial of the dose escalation study and was excluded, putting the final number of horses participating in trial three to *n* = 5. As the inflammation subsided over the following days, it was considered safe to include the horse in the subsequent multiple dose study. Oral application of the CBD product was well tolerated. Physical examinations showed no irregularities and mean assessments of CBCs, kidney and liver biomarkers remained within reference range throughout both trials in all horses (Table 2). Maximum white blood cell (WBC) count was 13.15 10^9^/L (reference range (RR): 5–10 10^9^/L). Values for BUN below RR were between 6.9–9.3 mg/dL (RR: 9.4–23.5 mg/dL) and for CREA between 0.8–0.9 mg/dL (RR: 0.9–1.5 mg/dL). GGT remained within RR in all samples. GOT was 387 IU/L in one horse (RR: 165–358 IU/L) after 7 days of treatment (Table 2).

**Table 2:** Mean ± standard deviation of WBC count, kidney and liver biomarkers during multiple administrations of CBD paste (3 mg/kg po) twice daily over two weeks with subsequent sample collection.

Parameter (RR)	Baseline	Day 7	Day 14	Day 21
WBC (5–10 10^9^/L)	9.0 ± 2.2	7.8 ± 1.6	7.9 ± 2.0	7.6 ± 1.9
Number of horses out of RR	*n* = 2/6	*n* = 1/6	*n* = 0/6	*n* = 1/6
BUN (9.4–23.5 mg/dL)	10.1 ± 1.1	11.0 ± 0.9	10.0 ± 1.0	11.3 ± 2.2
Number of horses out of RR	*n* = 2/6	*n* = 0/6	*n* = 1/6	*n* = 2/6
CREA (0.9–1.5 mg/dL)	1.0 ± 0.1	1.1 ± 0.2	1.0 ± 0.1	1.0 ± 0.1
Number of horses out of RR	*n* = 0/6	*n* = 1/6	*n* = 1/6	*n* = 1/6
GGT (10–50 IU/L)	22.3 ± 2.9	23.5 ± 4.8	23.0 ± 2.4	20.5 ± 3.3
Number of horses out of RR	*n* = 0/6	*n* = 0/6	*n* = 0/6	*n* = 0/6
GOT (165–358 IU/L)	290.2 ± 38.6	298.0 ± 47.5	288.8 ± 29.7	295.7 ± 21.8
Number of horses out of RR	*n* = 0/6	*n* = 1/6	*n* = 0/6	*n* = 0/6

The number of horses in each group with serum levels outside of RR are also reported. RR, reference range; WBC, white blood cell; BUN, blood urea nitrogen; CREA, creatinine; GGT, gamma-glutamyltransferase; GOT, glutamic oxaloacetic transaminase.

### 3.2. Pharmacokinetic analysis

#### 3.2.1. Non-compartmental analysis

##### 3.2.1.1. Dose escalation study

**Figure 1:**
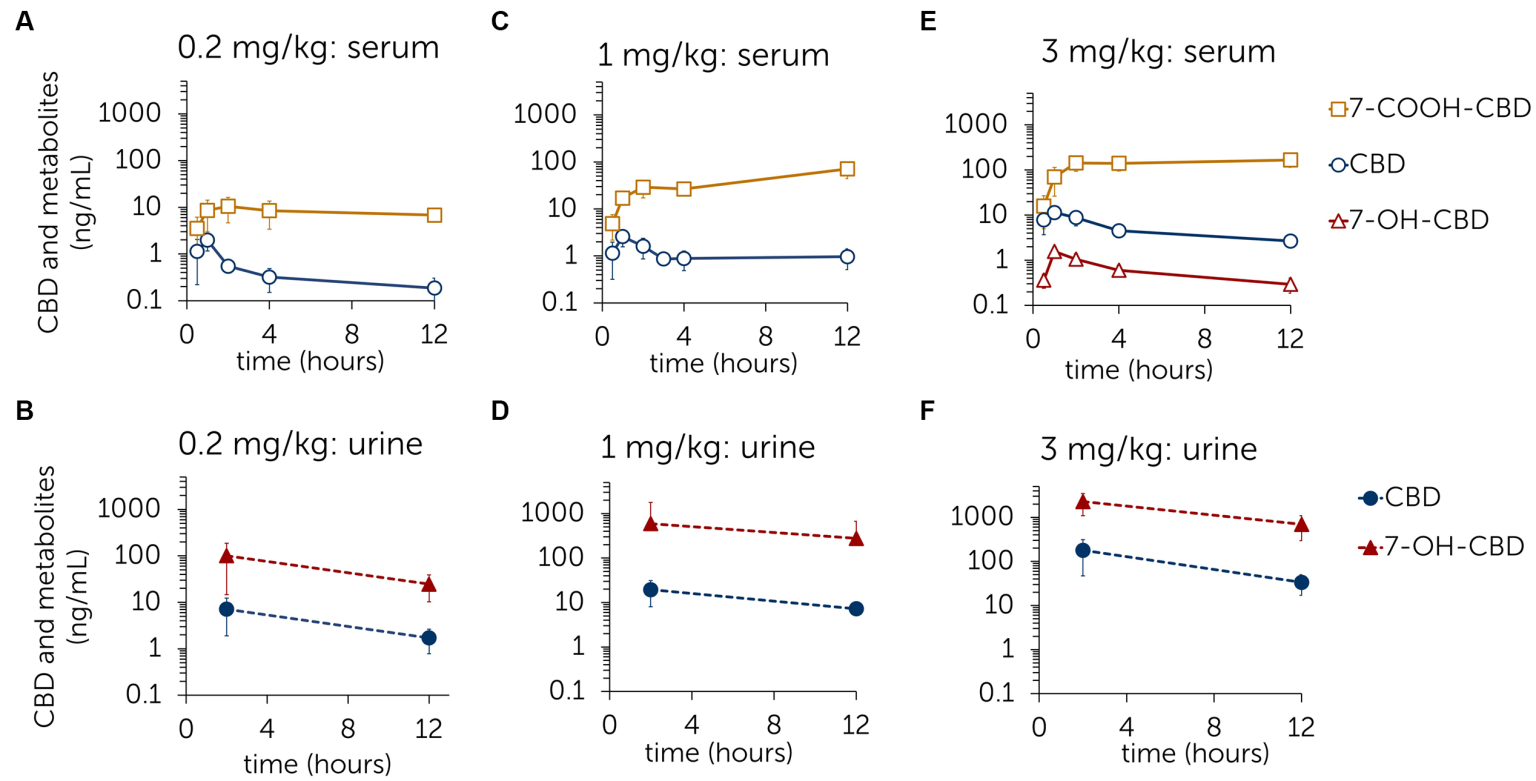
Mean ± standard deviation of serum and urine concentrations of cannabidiol (CBD) and the metabolites 7-hydroxy-cannabidiol (7-OH-CBD) and 7-carboxy-cannabidiol (7-COOH-CBD) after single oral administration of CBD paste in three different doses [0.2 mg/kg **(A,B)**; 1 mg/kg **(C,D)**; 3 mg/kg **(E,F)**].

Concentration curves with mean ± standard deviations of CBD and its main metabolites 7-COOH-CBD and 7-OH-CBD in serum and urine are shown in Figure 1. In the first trial (dose: 0.2 mg/kg), CBD and 7-COOH-CBD were found in serum and CBD and 7-OH-CBD were found in urine. In the second trial (dose: 1 mg/kg), CBD, 7-OH-CBD and 7-COOH-CBD were identified in serum, but 7-OH-CBD remained below the LLOQ. CBD, 7-OH-CBD, CBDA, CBDV and CBG were detected in urine with CBDA levels being below the LLOQ (Supplementary Figure S1). In the third trial (dose: 3 mg/kg), CBD, 7-OH-CBD and 7-COOH-CBD were identified in serum. In urine, CBD, 7-OH-CBD, CBDA, CBDV and CBG were detected (Figure 1; Supplementary Figure S1). CBDA levels were again below LLOQ. Table 3 presents the parameters AUC_0–12 h_, *C*_max_ and *t*_max_ assessed in the NCA and the AUC_0–12 h_ ratio between CBD and its metabolites 7-OH-CBD and 7-COOH-CBD. *C*_max_ and *t*_max_ could not be determined for 7-COOH-CBD, as the concentration curves have not decreased sufficiently by time point 12 h (Figure 1).

**Table 3:** Mean ± standard deviation of pharmacokinetic parameters for CBD and metabolites following single oral administrations of CBD paste during dose escalation study, derived from NCA.

Parameter	First trial (0.2 mg/kg, *n* = 3)	Second trial (1 mg/kg, *n* = 3)	Third trial (3 mg/kg, *n* = 5)
CBD			
AUC_0–12 h_ (h·ng/mL)	4.45 ± 2.52	15.46 ± 6.08	59.53 ± 13.54
*C*_max_ (ng/mL)	1.98 ± 0.99	2.58 ± 1.25	12.17 ± 2.08
*t*_max_ (hr)	1 ± 0	1 ± 0	1.1 ± 0.55
7-COOH-CBD			
AUC_0–12 h_ (h·ng/mL)	106.95 ± 65.68	571.02 ± 194.33	1768.38 ± 450.86
Ratio: $\frac{{\mathrm{AUC}}_{0-12\;h}\left(7-\mathrm{COOH}-\mathrm{CBD}\right)}{{\mathrm{AUC}}_{0-12\;h}\left(\mathrm{CBD}\right)}$	21.09 ± 3.19 (2109.15%)	38.78 ± 7.82 (3877.88%)	31.02 ± 6.38 (3102.13%)
7-OH-CBD			
AUC_0–12 h_ (h·ng/mL)	—	—	6.62 ± 1.86
Ratio: $\frac{{\mathrm{AUC}}_{0-12\;h}\left(7-\mathrm{OH}-\mathrm{CBD}\right)}{{\mathrm{AUC}}_{0-12\;h}\left(\mathrm{CBD}\right)}$	—	—	0.10 ± 0.03 (10.23%)
*C*_max_ (ng/mL)	—	—	1.42 ± 0.37
*t*_max_ (hr)	—	—	1.4 ± 0.55

NCA, non-compartmental analysis; CBD, cannabidiol; 7-COOH-CBD, 7-carboxy-cannabidiol; 7-OH-CBD, 7-hydroxy-cannabidiol; AUC_0–12 h_, area under the serum concentration-time curve (from time point 0 to 12 h); *C*_max_, maximum concentration; *t*_max_, time of maximum concentration.

##### 3.2.1.2. Multiple dose study

**Figure 2:**
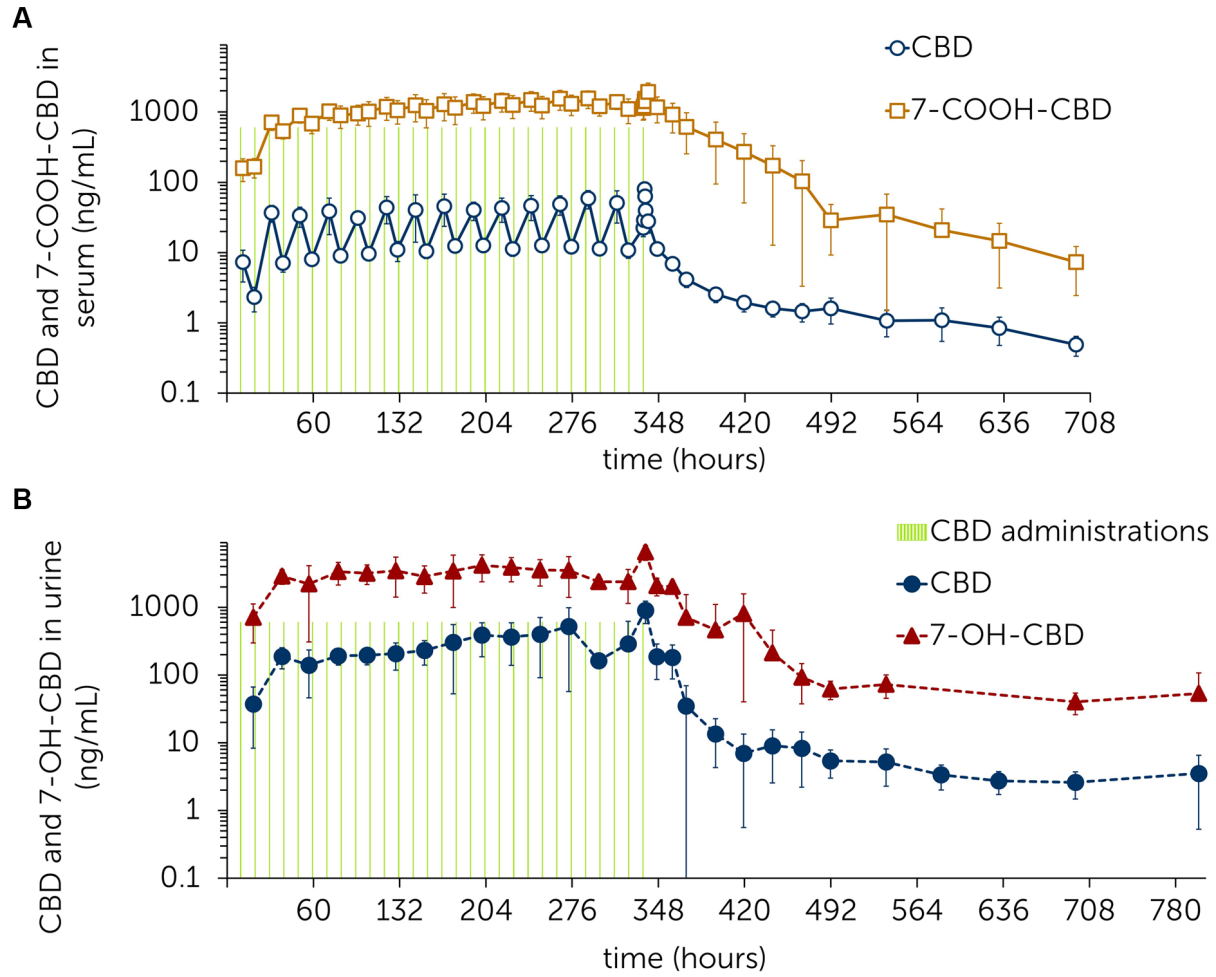
Mean ± standard deviation of serum **(A)** and urine **(B)** concentrations of cannabidiol (CBD) and the metabolites 7-hydroxy-cannabidiol (7-OH-CBD) and 7-carboxy-cannabidiol (7-COOH-CBD) following multiple administrations of CBD paste (3 mg/kg po) twice daily over 2 weeks with subsequent sample collection.

CBD, 7-OH-CBD, 7-COOH-CBD, CBDV, THC and OH-THC were identified in serum. 7-OH-CBD concentrations were below the LLOQ from 60 h after last CBD administration onwards (Figure 2). CBDV and THC were detected in concentrations around the LLOQ throughout the trial [*C*_max_(CBDV) = 0.39 ng/mL; *C*_max_(THC) = 0.70 ng/mL]. CBDV and THC values were below the LLOQ at 4 h and 12 h after last CBD administration. OH-THC concentrations remained mostly below the LLOQ except for the time points 202.5 h (0.26 ng/mL) and 314 h (0.27 ng/mL) (Supplementary Figure S2).

In urine, CBD, 7-OH-CBD, CBDA, CBDV and CBG were identified. CBDA concentrations fell below the LLOQ 36.5 h after the last CBD administration. CBG and CBDV values remained below the LLOQ 131 h and 248 h after the last CBD administration, respectively (Figure 2; Supplementary Figure S2).

The terminal half-life for CBD and 7-COOH-CBD in serum was calculated based on the last six time points (132–360 h) after the last CBD administration. For CBD, the terminal half-life was 161.29 ± 43.65 h and for 7-COOH-CBD, it was 79.85 ± 18.03 h.

#### 3.2.2. Population pharmacokinetic analysis

**Figure 3:**
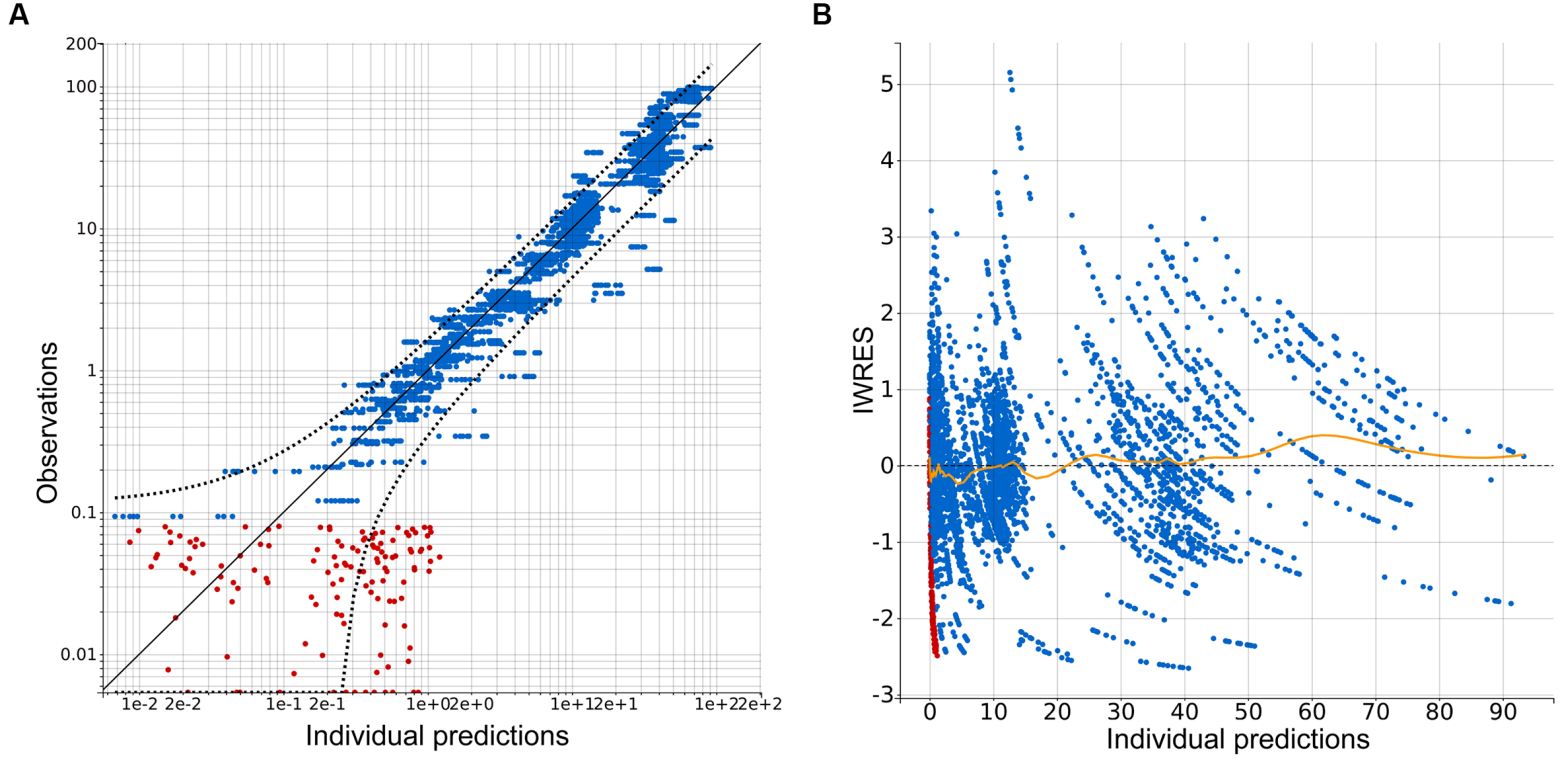
Diagnostic plots extracted from the three-compartment model following population pharmacokinetic analysis. **(A)** Plot of observations vs. individual predictions. Blue dots indicate observations, red dots indicate censored data, black line—identity line; dotted black line represents the 90% prediction interval. Outliers proportion was 10.54%. **(B)** Scatterplot of individual weighted residuals (IWRES) vs. individual predictions. Blue dots indicate observations, red dots indicate censored data, spline is marked with a yellow line.

**Figure 4:**
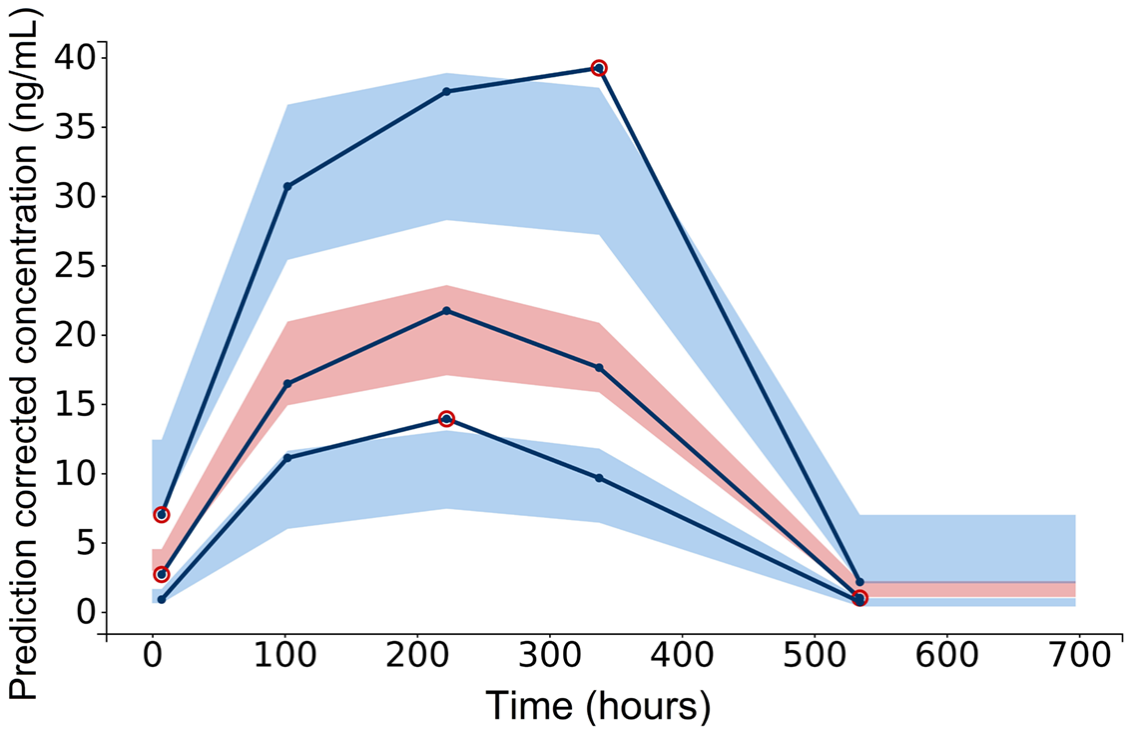
Diagnostic plot extracted from the three-compartment model following population pharmacokinetic analysis: visual predictive check for CBD concentrations in serum. Empirical data [10th, 50th (median) and 90th percentile] are marked by solid lines. Outlier dots are circled in red. Shaded areas mark the 90% confidence intervals for corrected prediction of the median (red) and the 10th and 90th percentile (blue).

**Figure 5:**
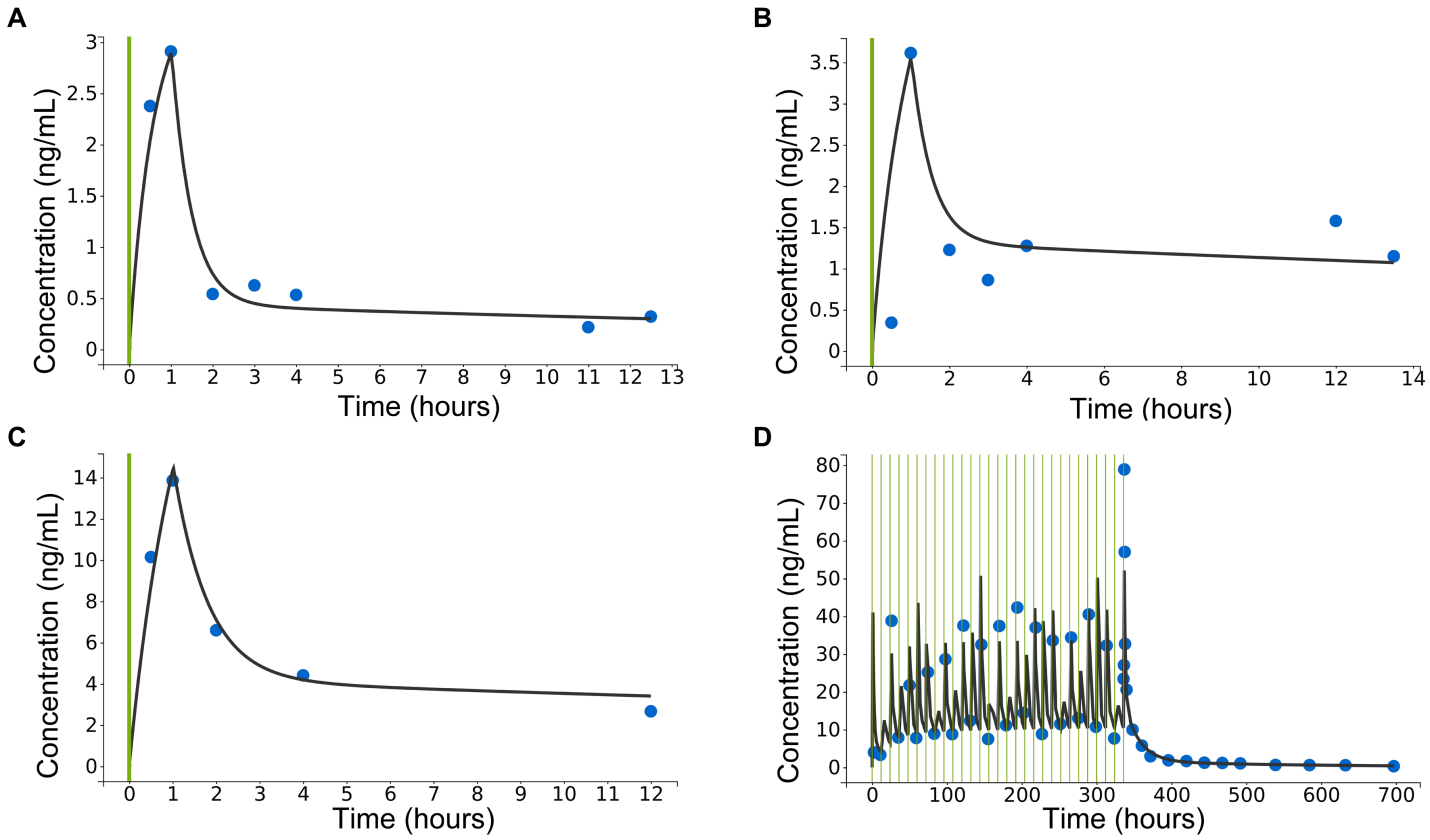
Diagnostic plots extracted from the three-compartment model following population pharmacokinetic analysis: exemplary individual predictions for concentrations of cannabidiol (CBD) in serum after single oral administration of CBD paste in three different doses [**(A)**: 0.2 mg/kg po; **(B)**: 1 mg/kg po; **(C)**: 3 mg/kg po], and **(D)**: following multiple administrations of CBD paste (3 mg/kg po) twice daily over 2 weeks with subsequent sample collection. Green lines represent CBD administrations, blue dots are observed data points and black lines are individual fits.

**Figure 6:**
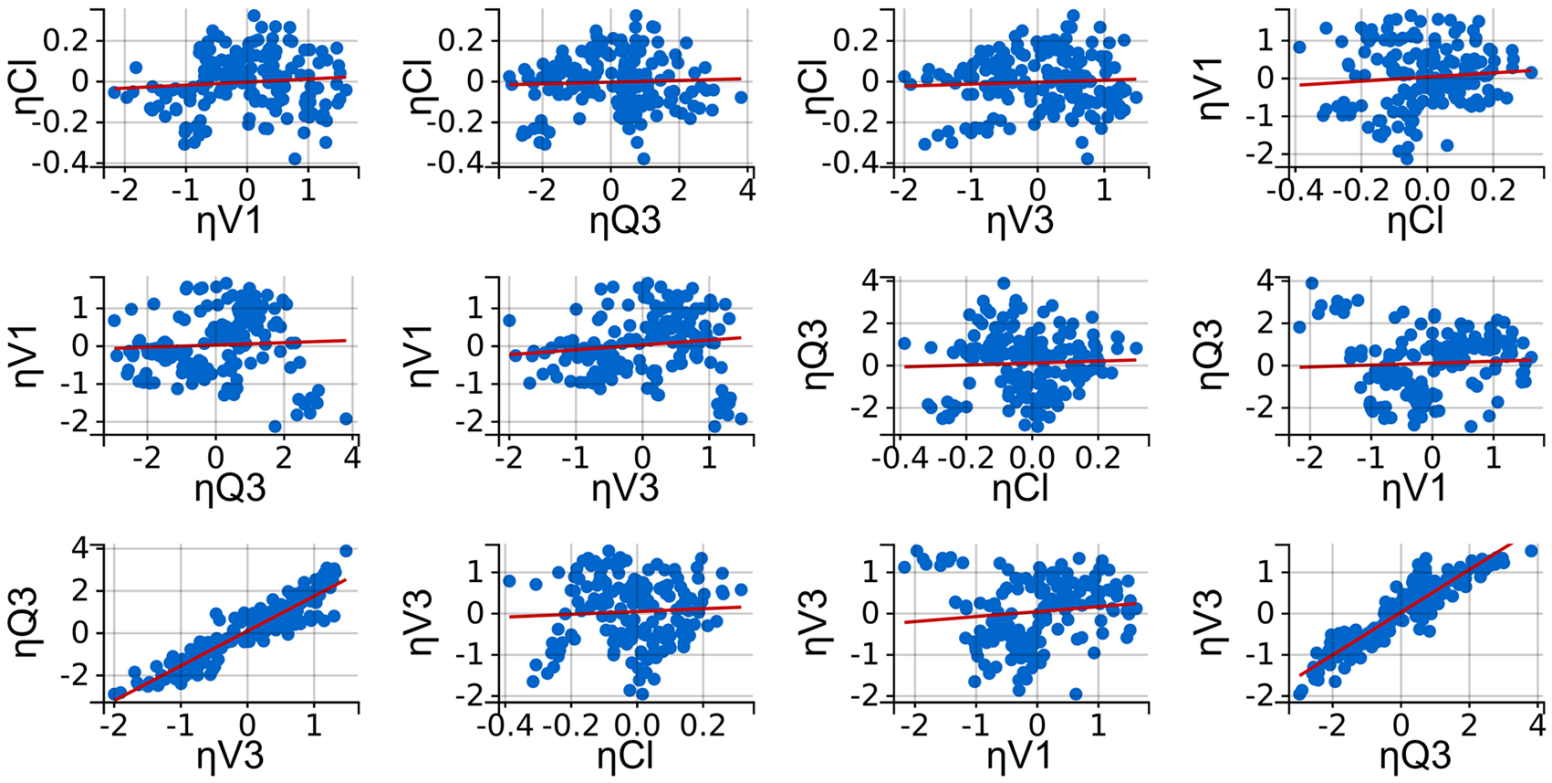
Diagnostic plot extracted from the three-compartment model following population pharmacokinetic analysis: Correlation plots of the random effects (*η_i_*). Correlation was applied when correlation coefficients were estimated to be high and met the threshold for inclusion (Pearson’s correlation test, *p* < 0.05). Linear regressions are presented as red lines.

A three-compartment model best described the pharmacokinetic properties of CBD in horses. Residual error was described through a combined 1 error model, containing a constant and proportional term. Numerical and graphical outputs were evaluated for GOF and predictive power. Diagnostic plots are shown in Figures 3–6. The visual predictive check (VPC) shows close prediction of median values (Figure 4). Empirical data for the 10th and 90th percentile are deviating from their respective confidence intervals (CI) at around 220 h and 350 h, respectively. Exemplary graphs depicting individual predictions are presented in Figure 5.

Inter-occasion variability (IOV) was not included as it was similar to the individual variability and, due to the relatively small number of subjects, led to a low precision of estimates. Profiles were therefore treated as separate individuals. Random effects were estimated for Cl/F, V1/F, Q3 and V3/F. For the other parameters, the population value was used as the random effects were converging to zero and were insufficiently assessed in all individuals. Correlating V3/F and Q3 further improved the fit of the model (Figure 6).

Table 4 presents the final pharmacokinetic parameters derived through the population pharmacokinetic approach. The low relative standard error (RSE) values confirm accurate assessment for the population parameter estimates. The low eigenvalue ratio (29.07, derived from the Fisher information matrix) and low shrinkage (< 20%, see Table 4) indicate that the model was not over-parameterized. The values for volume of distribution in the central (V1/F) and peripheral compartments (V2/F and V3/F) suggest a very high distribution of CBD as well as retention in tissues. The estimation of convergence accounts for the model’s robustness.

**Table 4:** Population pharmacokinetic parameters of orally administered CBD paste in four different equine trials.

	Population value	SE	RSE (%)	Omega	SE	RSE (%)	Shrinkage (%)
Population parameter estimates (unit)							
Tk0 (h)	1.02	0.11	10.5	—	—	—	—
Cl/F (L/h/kg)	10.75	0.7	6.53	0.15	0.049	33.6	15.1
V1/F (L/kg)	77.13	20.11	26.1	0.83	0.18	22.1	2.27
Q2 (L/h/kg)	1.35	0.14	10.2	—	—	—	—
V2/F (L/kg)	313.17	50.63	16.2	—	—	—	—
Q3 (L/h/kg)	38.23	15.72	41.1	1.48	0.47	31.8	9.11
V3/F (L/kg)	241.98	67.77	28.0	0.85	0.24	28.0	12.9
Residual error							
a	0.07	0.021	29.8	—	—	—	—
b	0.33	0.016	5.04	—	—	—	—

Data derived from three separate trials with single doses of 0.2 mg/kg (administered to *n* = 3 horses), 1 mg/kg (*n* = 3) and 3 mg/kg (*n* = 5) and a multiple dose study with a dose of 3 mg/kg administered twice daily over 15 days (*n* = 6). CBD, cannabidiol; SE, standard error; RSE, relative standard error, Tk0, duration of the zero-order absorption; Cl/F, total body clearance; V1/F, volume of distribution in the central compartment; V2/F, volume of distribution in the first peripheral compartment; V3/F, volume of distribution in the second peripheral compartment; Q2, clearance between V1 and V2; Q3, clearance between V1 and V3; F, bioavailability.

Bodyweight as an added covariate did not show any effect on the pharmacokinetic parameters and was excluded from the final model.

AUC_0–12 h_ as an additional output and *C*_max_ and *t*_max_ (extracted from individual fits) are presented in Table 5. Values are shown in relation to the parameters derived from the NCA (Table 3).

**Table 5:** Mean ± standard deviation of pharmacokinetic parameters for CBD and metabolites following single oral administrations of CBD paste during the dose escalation study, derived from the individual fits of the population pharmacokinetic model.

	First trial (0.2 mg/kg, *n* = 3)	Second trial (1 mg/kg, *n* = 3)	Third trial (3 mg/kg, *n* = 5)
AUC_0–12 h_ (h·ng/mL)	4.99 ± 1.56	13.64 ± 5.33	58.56 ± 12.98
*C*_max_ (ng/mL)	1.82 ± 0.83	3.10 ± 1.27	14.61 ± 5.08
*t*_max_ (hr)	1.01 ± 0.01	1.02 ± 0.03	1.02 ± 0.01
Ratio: $\frac{\;\mathrm{Parameter}\;\left({\mathrm{CBD}}_{\mathrm{Pop}\_\mathrm{PK}}\right)}{\mathrm{Parameter}\;\left({\mathrm{CBD}}_{\mathrm{NCA}}\right)}$			
$\;\;\;\frac{{\mathrm{AUC}}_{0-12\;h}\left({\mathrm{CBD}}_{\mathrm{Pop}\_\mathrm{PK}}\right)\;}{{\mathrm{AUC}}_{0-12\;h}\left({\mathrm{CBD}}_{\mathrm{NCA}}\right)}$	1.20 ± 0.24	0.86 ± 0.11	0.98 ± 0.09
$\;\;\;\frac{C_{\max}\left({\mathrm{CBD}}_{\mathrm{Pop}\_\mathrm{PK}}\right)\;}{C_{\max}\left({\mathrm{CBD}}_{\mathrm{NCA}}\right)}$	0.92 ± 0.08	1.21 ± 0.21	1.18 ± 0.29
$\;\;\;\frac{t_{\max}\left({\mathrm{CBD}}_{\mathrm{Pop}\_\mathrm{PK}}\right)\;}{t_{\max}\left({\mathrm{CBD}}_{\mathrm{NCA}}\right)}$	1.01 ± 0.01	1.02 ± 0.03	1.12 ± 0.52

Values are presented as ratios to the parameters derived from the non-compartmental analysis (Table 3). CBD, cannabidiol; AUC_0–12 h_, area under the serum concentration-time curve from time point 0 to 12 h; *C*_max_, maximum concentration, *t*_max_, time of maximum concentration; NCA parameters, parameters derived from non-compartmental analysis; CBD_Pop_PK_, parameter for CBD derived through population pharmacokinetics; CBD_NCA_, parameter for CBD derived through non-compartmental analysis.

To calculate the accumulation ratio (AR), *C*_max_ from each day of the multiple dose study was summarized to a mean of 38.39 ± 8.89 ng/mL. Mean *C*_max_ from trial 3 of the dose escalation study was 14.61 ± 5.08 ng/mL. AR was therefore 2.63.

#### 3.2.3. Dose proportionality

The power model equation revealed the *β* value for the NCA parameter AUC_0–12 h_ to be 0.99 and for *C*_max_ to be 0.72. For the population pharmacokinetic parameters, the *β* value for AUC_0–12 h_ was 0.93 and 0.80 for *C*_max_. As the individual values were pooled for this approach, the inter-individual variability through a CI was not determined.

An ANOVA with a post-hoc Tukey test identified a significant difference between the dose-normalized *C*_max_ obtained from NCA between trial 1 (0.2 mg/kg) and trial 2 (1 mg/kg) (*p* = 0.014). Trials 2 and 3 (3 mg/kg), and trials 1 and 3 showed no statistically significant differences (*p* = 0.334, *p* = 0.123). Similarly, there were no statistically significant differences between the other pharmacokinetic parameters.

### 3.3. Application to medication control

**Figure 7:**
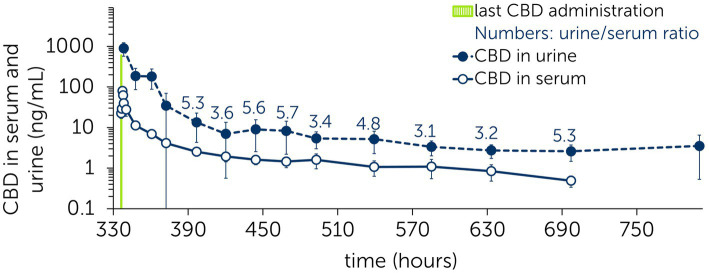
Mean ± standard deviation of serum and urine concentrations of cannabidiol (CBD) during the elimination phase. Last CBD administration (dose: 3 mg/kg) to six horses at time point 336 h following multiple administrations of CBD paste (3 mg/kg po) twice daily over 2 weeks. Numbers present the urine/serum ratio between respective time points.

Between 60 to 360 h after the last CBD administration in the multiple dose study, a pseudo-equilibrium condition was reached (Figure 7) (47, 48). The steady-state urine to serum concentration ratio (Rss) was calculated from the mean concentration values: Rss = 4.45 ± 1.04.

## 4. Discussion

Investigation of the pharmacokinetic properties of CBD following repeated oral administration identified a rapid increase of the CBD serum concentration with an extended elimination phase of CBD and its metabolites. These findings indicate an extensive metabolism of CBD with prolonged tissue retention.

The oral administration of CBD paste was well-tolerated by all horses in the current study and side effects such as gastrointestinal intolerance were not observed. A previous study reported mildly elevated liver enzymes after multiple oral administrations of a CBD-infused oil (1 mg/kg and 3 mg/kg) in horses (30). Another study reported decreased creatinine levels and higher gamma-glutamyltransferase levels, although still within normal reference range (49). In this study, only occasional, slight shifts out of RR without associated clinical signs were observed in WBC count, kidney and liver biomarkers.

Like in other equine and small animal investigations, the pharmacokinetic analysis showed a rapid increase of CBD in serum following oral administration (15, 16, 28, 29, 50–55). The values for *C*_max_ were similar to those calculated in other studies (28–31, 33). In contrast, the AUC_0–12 h_ values obtained here differ significantly. This is caused by the fact that in the previous studies AUC were determined over longer time periods (up to 264 h) (28–31, 33). The AUC_0–12 h_ values reported for the single dose part of the current study are much lower as the time dimension of this parameter is terminated at 12 h. It was not possible to credibly determine relative bioavailability for the used formulation. This would require calculating AUC_0–∞_ and compare it with the results of previously published studies. As for the single dose administration, the terminal portion of the curve was not sufficiently captured to assess AUC_0–∞_.

A long elimination phase for CBD was shown during the multiple dose study (Figure 2). Based on the visual inspection of the individual log-linear concentration-time profiles, the terminal phase of elimination started approx. 132 h after the last CBD administration. Therefore, only the following data-points were used for the calculation of the elimination half-life. As previous studies have derived the terminal half-life from earlier time points, values are difficult to compare (28–31, 33). The very long elimination phase of CBD suggests a high volume of distribution into different tissue compartments.

Previous studies hypothesized, that CBD is subject to a high first pass effect with a considerable pre-systemic metabolism in the liver (29, 33, 56). The extensive metabolism of CBD into 7-COOH-CBD is mirrored by the high ratio of their AUC_0–12 h_ (Table 3). In comparison, the AUC_0–12 h_ ratio between CBD and 7-OH-CBD is substantially lower. To the best of the authors knowledge, research detailing the exact steps of CBD metabolism in horses is currently not available. In humans, 7-OH-CBD is further metabolized to 7-COOH-CBD (57, 58). Based on this information, the low serum value of 7-OH-CBD in the current study may be explained by the partial metabolism into 7-COOH-CBD. In line with other reports, higher concentrations of 7-OH-CBD were detected in urine (29). Further research investigating the exact metabolic pathway of CBD in horses following oral administration would be of great interest.

For data derived from the NCA and the population pharmacokinetic approach, CBD ratios for AUC_0–12 h_, *C*_max_ and *t*_max_ were close to 1, confirming that the individual fits calculated in the NLME model are close to the actual concentrations measured (Table 5).

Values for volumes of distribution and clearance [both over bioavailability (F)] were derived through the population pharmacokinetic analysis. Although the study design did not include intravenous administration to precisely estimate the true clearance and volumes of distribution, the application of NLME modelling allowed the pooling of data into a single robust model, despite different study designs (single vs. multiple administrations) and dose levels. Volumes of distribution over F were high in the central and the two peripheral compartments (Table 4). Other studies in horses and dogs describe similar values based on non-compartmental analysis, even though doses and study protocols differ slightly (28, 51). Values are especially high for V2/F and V3/F in the current study, suggesting a very high distribution and tissue retention of CBD. This observation is further supported by the low inter-compartmental clearance value Q2 (1.35 L/h/kg) between V1 and V2. One reason might be the lipophilic properties of CBD, as confirmed by several canine and human studies (5, 10, 38). The high volumes of distribution could however be misleading, as the population pharmacokinetic model does not account for the extensive metabolism of CDB to 7-COOH-CBD. The authors chose to exclude the additional metabolite data out of the NLME modelling, as its inclusion and the subsequent classification of CBD as a parent drug did not produce a satisfying and stable model. The relatively small sample size and the lack of data for intravenous administration necessitated the choice of a simpler but much more stable model that met all the goodness-of-fit criteria.

The estimated clearance value of 10.75 L/h/kg is comparable to one study (33), but lower than the results from other equine studies that were also obtained using oral data with an unknown F (29, 30). Comparing clearance values with those from other species proved to be difficult, as very few reports exist and values are declared in L/h instead of L/h/kg (51, 56). One study reports a very high variance for clearance of CBD and its metabolites in dogs (59).

Considering all species, only few reports compare oral and intravenous administrations of CBD to calculate *F*. *F* has been described to be 7.92% and 14% in horses, putting it in a similar range with findings in humans (6%) and dogs (13%–22.28%) (31, 33, 51, 56, 60). The low *F* values further confirm the high first-pass-effect of CBD with extensive pre-systemic metabolism and a high liver extraction ratio, as described in humans (72%) (29, 56).

The visual predictive check of the population pharmacokinetic analysis shows good agreement with the median values, but there is a noticeable deviation of the 10th and the 90th percentile’s empirical data from the 10% and 90% CI at approximately 220 and 350 h after the first CBD administration (Figure 4). These deviations are likely caused by the differing concentration values of CBD in serum in one horse. This particular horse showed consistently higher values than the median. This may have been caused by interindividual variability or over-dosing of the CBD paste due to variation of the horse’s bodyweight. The authors decided not to exclude this horse from the dataset, as the other values were not affected by the described deviation. Moreover, such high variability in the internal exposure is not uncommon for drugs with low bioavailability, therefore the authors believe that this dataset may reflect the real-life situation well.

As the CBD product used in this study was extracted from the cannabis plant (*Cannabis sativa*), further phytocannabinoids were identified during the serum and urine analysis. Values for CBDV and THC in serum were very low throughout the study and reached levels just above LLOQ. In urine, CBDV and CBG were detected in higher concentrations. There is very little information available on the potential effects of these phytocannabinoids. One study reports CBDV to have an anti-convulsant effect in mice and rats (61). CBG’s influence on pain perception has been tested in mouse models (62, 63) and its pharmacokinetic properties have recently been described in dogs (64). Another study showed that CBG decreases the intraocular pressure in cats (65). The potential therapeutic use of CBG for the treatment of human diseases like multiple sclerosis has additionally been suggested (66).

During the multiple dose study, the steady state for CBD was reached at day 2 (Figure 2). The accumulation ratio (AR) under steady state for CBD in serum was 2.63. In humans, an AR of 2–5 is considered to indicate moderate drug accumulation (67). The time it takes to eliminate CBD from the bloodstream is therefore moderately long compared to the dosing interval (12 h). This observation might be helpful in establishing dosing patterns or time points for maximum efficacy. Concentration values in urine are less stable but are also showing fair consistency from day 2 onwards. As urine samples were collected as spot samples, values must be evaluated with caution.

The dose proportionality evaluated with an ANOVA did not identify any statistically significant differences in the dose-normalized parameters between trials, except for *C*_max_ obtained from the NCA between trial 1 (dose: 0.2 mg/kg) and 2 (dose: 1 mg/kg). Since *C*_max_ between trial 1 and trial 3 (dose: 3 mg/kg), and trials 2 and 3 did not differ significantly, this variability might be explained in part by the low bioavailability and small sample size in the dose escalation study. In the power model, *C*_max_ from the NCA had the lowest *β* value (0.72), confirming the variability and therefore possible lack of proportionality as seen in the ANOVA. *β* values for AUC_0–12 h_ were very close to 1, suggesting that CBD administered as a paste within the studied dose range leads to a dose proportional exposure with the extent of absorption remaining unchanged. On the other hand, the rate of absorption appears to decrease with higher doses as the increase for *C*_max_ becomes less linear (exemplified by the comparatively small *β* values). This observation may further support the choice of zero-order absorption as a model parameter in the population pharmacokinetic analysis. However, the small number of individuals within the specific dose groups and the high variability in exposure reduce the statistical significance of these results.

Graphical illustration shows that CBD concentrations in serum and urine achieve a pseudo-equilibrium condition during the elimination phase (Figure 7) (48). The values exemplify that CBD concentrations detected in serum can be translated to residual concentrations in urine by the calculated Rss. Whether these residual concentrations influence a horse’s performance and must be subject to medication control, remains unclear. Specific cut-off values for a drug can be defined through a nonexperimental approach, where irrelevant drug plasma concentrations (IPC) and irrelevant drug urine concentrations (IUC) are calculated (47). IPC and IUC are based on the average effective plasma concentration (EPC), which is derived from the standard dose (per dosing interval) and bioavailability. As no standard dose with a proven effect for CBD in horses has been defined so far, EPC, IPC and IUC were not calculated in the current study.

Limitations of the study include the lacking assessment of the inter-occasion variability (IOV) due to the small sample size and testing of only one CBD product through only one route of administration. Further studies may evaluate varying CBD doses administered intravenously to obtain precise estimates for clearance, volumes of distribution and bioavailability, and to gain a better understanding of CBD’s metabolism.

## 5. Conclusion

This study confirms the extensive metabolism of CBD and suggests a prolonged retainment in tissues resulting in the extended elimination phase of CBD and its metabolites. The oral administration of CBD paste proved to be well-tolerated and did not cause any side effects at a maximum dose of 3 mg/kg following oral administrations twice daily over 2 weeks. A population pharmacokinetic model pooling data from both single and multiple dose studies has been successfully developed. Whilst the steady-state urine to serum concentration ratio (Rss) was defined, future research analyzing the effect of CBD on behavioral parameters and anti-inflammatory responses are required. Once an effective therapeutic dose is established, specific cut-off values for medication control may be established further. Until then, the administration of CBD products to sport horses should be treated with caution.

## Data availability statement

The raw data supporting the conclusions of this article will be made available by the authors, without undue reservation.

## Ethics statement

The animal study was reviewed and approved by the competent authority for licensing and notification procedures for animal experiments (LAVG) in Brandenburg, Germany (AZ: 2347-12-2021).

## Author contributions

FE and AE were involved in all parts of the project. CL, WB, MM, and IS contributed to study design, planning of the project, and data analysis. FE and NB were responsible for study execution including animal handling and data collection. AW performed the drug assays under supervision of MT, MM, and IS. BP and FE performed the pharmacokinetic analyses. FE wrote the manuscript. All authors contributed to the article and approved the submitted version.

## Funding

The study was funded by the Freie Universität Berlin, the German Equestrian Federation (FN), and Herosan healthcare GmbH. Herosan healthcare GmbH was not involved in the study design, collection, analysis, interpretation of data, the writing of this article or the decision to submit it for publication. We acknowledge support by the Open Access Publication Fund of the Freie Universität Berlin.

## Conflict of interest

The authors declare that the research was conducted in the absence of any commercial or financial relationships that could be construed as a potential conflict of interest.

## Publisher’s note

All claims expressed in this article are solely those of the authors and do not necessarily represent those of their affiliated organizations, or those of the publisher, the editors and the reviewers. Any product that may be evaluated in this article, or claim that may be made by its manufacturer, is not guaranteed or endorsed by the publisher.
