# Supplementary material for: Pharmacokinetic modelling of orally administered cannabidiol and implications for medication control in horses
Source: Front Vet Sci. 2023 Aug 9;10:1234551. doi: 10.3389/fvets.2023.1234551 (PMC10445762; doi:10.3389/fvets.2023.1234551)
Supplement: Supplementary file 1 [file Data_Sheet_1.PDF]

# Supplementary Material

## 1 Supplementary Data

### 1.1 Preparation of urine samples for cannabinoid analysis

Cannabinoids were extracted from 5 mL urine by liquid/liquid extraction after addition of 50 µL of the internal standard mix (D3-CBD, D3-THC, D3-OH-THC, D3-COOH-THC, each at 1 µg/mL in methanol) and after hydrolysis of the phase-II metabolites. For the hydrolysis, samples were adjusted to pH 7 with 1 mL of 0.8 M phosphate buffer and then incubated after addition of 50 µL β-glucuronidase from *E. coli* at 50 °C for 1 h. Six mL of n-pentane were added and the mixture was shaken for 20 min and subsequently centrifuged at 600 g for 5 min. The n-pentane layer was separated and evaporated to dryness under reduced pressure. The dry residue was derivatized with 80 µL MSTFA/NH4I/ethanethiol 1000:2:3 (v:w:v) for 30 min at 80 °C and 6 µL were injected onto the gas chromatograph/mass spectrometry instrument.

### 1.2 Preparation of serum samples for cannabinoid analysis

Cannabinoids were extracted from 2 mL serum by liquid/liquid extraction after addition of 20 µL of the internal standard mix (D3-CBD, D3-THC, D3-OH-THC, D9-COOH-THC, each at 1 µg/mL in methanol). The mixture was adjusted to pH 5.2 with 100 µL of 4 M sodium acetate buffer. Five mL of a 50:50 (v:v) mixture of n-pentane and tert-butyl-methyl-ether were added and the mixture was shaken for 20 min and subsequently centrifuged at 600 g for 5 min. The organic layer was separated and evaporated to dryness under reduced pressure. The dry residue was derivatized with 80 µL MSTFA/NH4I/ethanethiol 1000:2:3 (v:w:v) for 30 min at 80 °C and 6 µL were injected onto the gas chromatograph/mass spectrometry instrument.

### 1.3 Gas chromatography/tandem mass spectrometry (GC/MS/MS)

Analyses were performed using a Thermo Scientific TSQ 8000EVO tandem mass spectrometer coupled to a Thermo Scientific Trace 1310 gas chromatograph. A J&W Ultra 1 column (length 17 m, I.D. 0.2 mm, film thickness 0.11 µm) was employed, and helium was used as carrier gas at a constant pressure of 17.6 psi. An aliquot of 6 µL of the sample extract was injected into the GC/MS/MS system, which was operated in split mode (1:10). The GC temperature was ramped as follows: initial temperature = 157 °C, program rate = 20 °C/min to 325 °C, constant temperature = 325 °C for 1 min. The injection port and transfer line were heated to 300 °C. The trimethylsilylated analytes were measured using selected reaction monitoring (SRM) after electron ionisation (EI) and collision induced dissociation (CID) with argon as collision gas. The diagnostic ion transitions (listed as m/z), retention times (RT) and collision energies (CE) for each compound are presented in Table 1S.

Table 1S: Diagnostic ion transitions (specified as m/z), retention times (RT in min) and collision energies (CE in V) for each analyte and the corresponding internal standards (ISTD).

| **Cannabinoid** | **RT** | **m/z** | **(CE)** | **ISTD** | **RT** | **m/z (CE)** | |
|---|---|---|---|---|---|---|---|
| CBD | 4.33 | 390/301 | (8) | D3-CBD | 4.32 | 393/304 | (10) |
| CBDA | 5.45 | 491/133 | (29) | D9-Carboxy-THC | 6.28 | 380/314 | (10) |
| CBDV | 3.63 | 362/273 | (7) | D3-Hydroxy-THC | 5.78 | 374/292 | (13) |
| CBG | 5.03 | 337/321 | (9) | D3-CBD | 4.32 | 393/304 | (10) |
| 7-COOH-CBD | 5.70 | 443/119 | (14) | D9-Carboxy-THC | 6.28 | 380/314 | (10) |
| 7-OH-CBD | 5.36 | 443/337 | (9) | D3-Hydroxy-THC | 5.78 | 374/292 | (13) |
| COOH-THC | 6.30 | 371/305 | (12) | D9-Carboxy-THC | 6.28 | 380/314 | (10) |
| OH-THC | 5.79 | 371/305 | (7) | D3-Hydroxy-THC | 5.78 | 374/292 | (13) |
| THC | 4.72 | 389/371 | (11) | D3-THC | 4.71 | 389/374 | (10) |

Abbreviations: CBD, cannabidiol; CBDA, cannabidiolic acid; CBDV, cannabidivarin; CBG, cannabigerol; 7-COOH-CBD, 7-carboxy-cannabidiol; 7-OH-CBD, 7-hydroxy-cannabidiol; THC, Δ9-tetrahydrocannabinol; COOH-THC, 11-nor-9-carboxy-Δ9-tetrahydrocannabinol; OH-THC, 11-hydroxy-Δ9-tetrahydrocannabinol.

## 2 Supplementary Figures

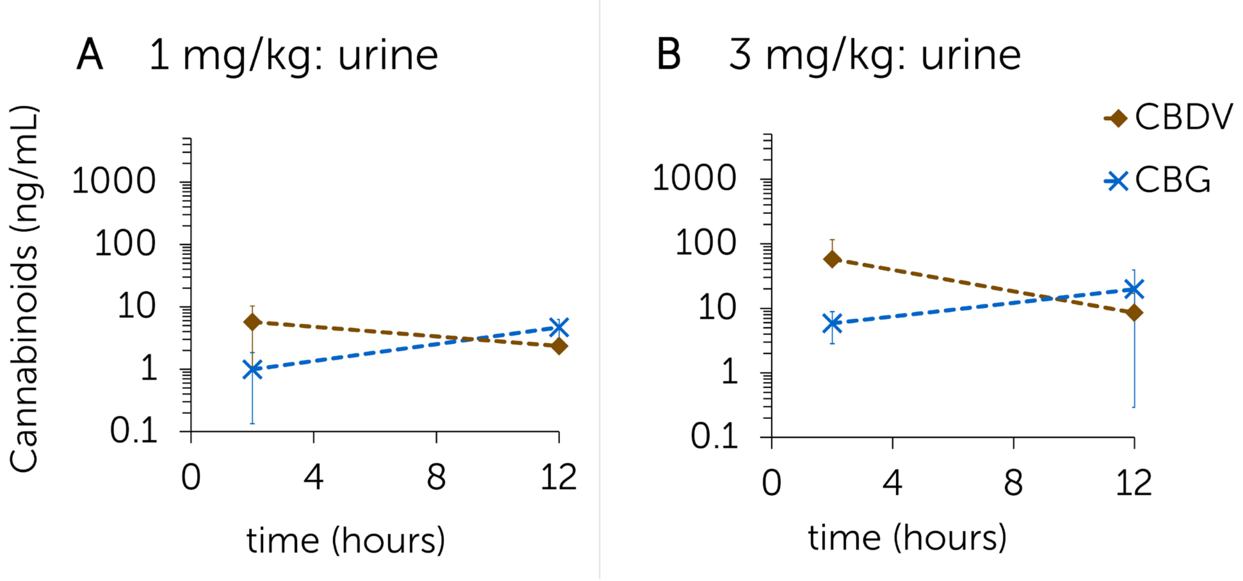


Supplementary Figure 1S: Mean ± standard deviation of cannabidivarin (CBD) and cannabigerol (CBG) in urine after single oral administration of CBD paste in two different doses (1mg/kg po (A); 3mg/kg po (B)).

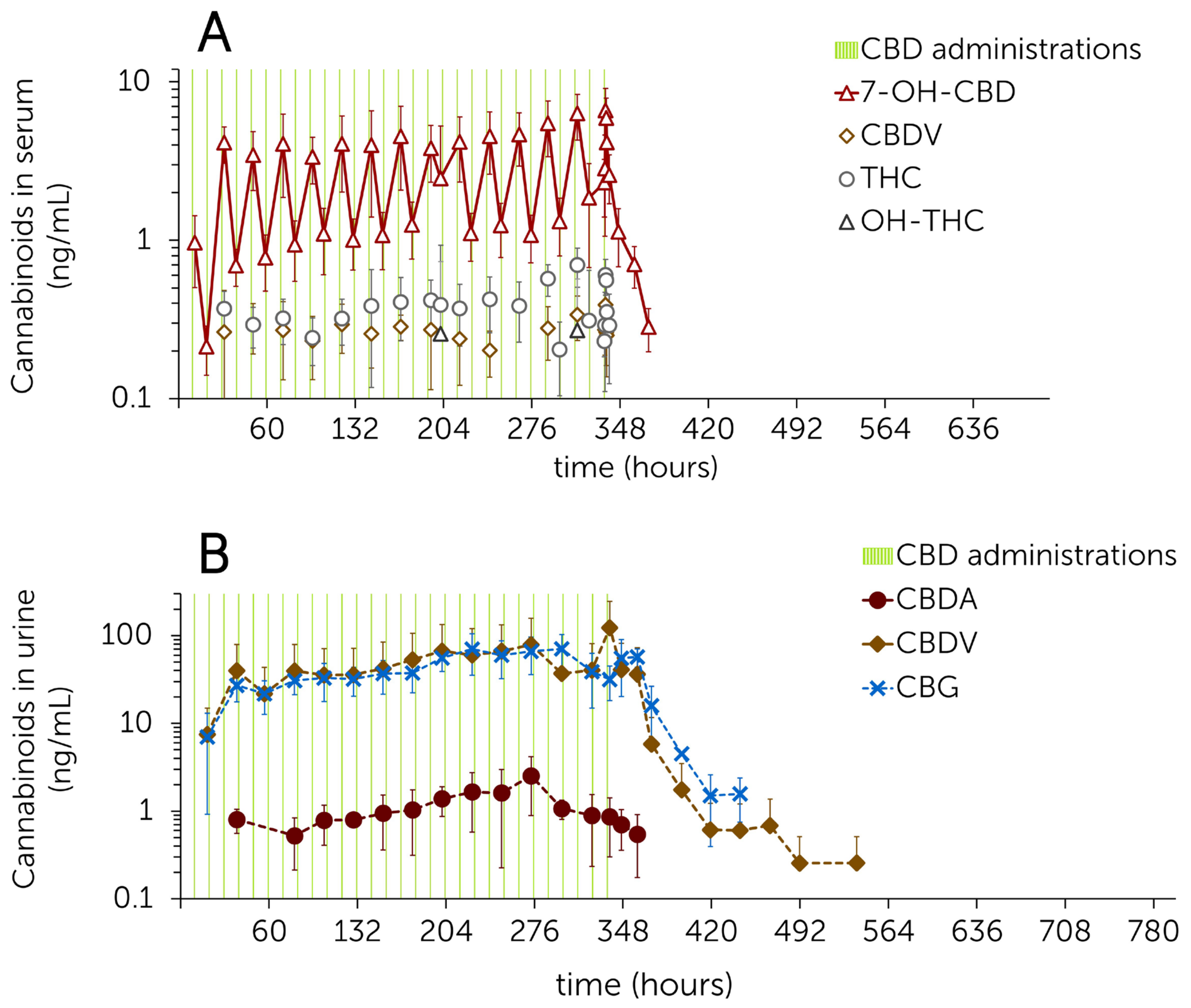


Supplementary Figure 2S: Mean ± standard deviation of the following cannabinoid concentrations: 7-hydroxy-cannabidiol (7-OH-CBD), cannabidivarin (CBDV), Δ9-tetrahydrocannabinol (THC) and 11-hydroxy-THC (OH-THC) in serum (A), and cannabidiolic acid (CBDA), CBDV and cannabigerol (CBG) in urine (B) following multiple administrations of CBD paste (3 mg/kg po) twice daily over two weeks with subsequent sample collection.
